# Changes in adhesion molecules: β-catenin, E-cadherin and Galectin-3 in cells of testicular seminoma

**DOI:** 10.3389/fonc.2023.1269637

**Published:** 2023-12-08

**Authors:** Grzegorz Młynarczyk, Natalia Domian, Irena Kasacka

**Affiliations:** ^1^ Department of Urology, Medical University of Białystok, Bialystok, Poland; ^2^ Department of Histology and Cytophysiology, Medical University of Białystok, Bialystok, Poland

**Keywords:** β-catenin, E-cadherin, galectin-3, testicular cancer, seminoma

## Abstract

**Introduction:**

The most common testicular tumors are seminomas. They are characterized by rapid growth and a very high potential for metastasis to other organs. Mutual interactions of tumor cells play an important role in the invasiveness and metastatic capacity, in which complexes of adhesion proteins play a special role. There is a lack of studies on changes in these molecules and their behaviour in testicular cancer. The aim of the study was immunohistochemical identification and evalutaion of adhesive molecules β-catenin, E-cadherin, galectin-3 in testicular cancer – seminoma.

**Methods:**

Tests were performed on sections of testicular cancer – seminoma in comparison with unchanged tissue samples as a control. Material was taken from 30 patients who underwent orchiectomy. Immunohistochemistry and PCR were used to identify β-catenin, E-cadherin and galectin-3 and gene expression.

**Results:**

Immunoreactivity and expression of β-catenin and E-cadherin in seminomas were markedly decreased compared to non-cancerous testicular tissue. Galectin-3 immunoreactivity was found in both control and cancerous tissue, but in different location. In non-cancerous tissue, it was localized in the cytoplasm of the cells of the seminiferous tubules, in seminomas it was localized mainly in the endothelium. The expression of the Lgals3 gene encoding galectin-3 in seminomas was slightl higher in relation to the tissue unchanged by the carcinogenetic process.

**Conclusions:**

The results of the study suggest a significant role of β-catenin, E-cadherin and galectin-3 in the carcinogenesis of seminomas and may indicate new aspects of the patomechanism of seminomas formation, and thus time lead to better understand the biology of these tumors.

## 1 Introduction

In recent decades, there has been an increase in the incidence of testicular cancer (1–3), which accounts for about 5% of all urological cancers. The most common histopathological form of testicular cancers are seminomas (4–6). They account for about a third of all testicular germ cell tumors. The disease probably has its origin in fetal life as testicular dysgenesis syndrome (TDS). The exact pathomechanism of the development of seminomas is not known. The most likely theory is inhibition of gonocyte maturation, but the exact pathogenesis is still unknown. Therefore, further research on the pathomechanisms of this tumor may prove to be very important in the diagnosis and effective therapy.

Adhesion molecules, which are involved in the organization, differentiation and proliferation of cells, play a very important role in the proper functioning of cells and tissues. Against this background, they seem to play a key role in tumorigenesis. Disturbances in the interaction between cells and the components of the extracellular matrix lead to a loss of cohesion and facilitate cell migration, contributing to the formation of metastases. In addition, adhesion proteins allow cancer cells to penetrate the wall of blood vessels and stimulate angiogenesis, which affects the formation of metastases in sometimes distant organs. Recent studies indicate that adhesion molecules play an important role in various stages of malignant tumor development, stimulate primary tumor growth and, through intracellular signal transduction mechanisms, enable cancer cells to migrate through the blood vessel wall and metastasize (7, 8).

One of the families of adhesion proteins are cadherins, which play a key role in securing intercellular contacts. Test results for several cancers (breast cancer, colorectal cancer, bladder, esophagus) showed poor prognosis and worse course of the disease with reduced expression of E-cadherin and catenins (9–13).

Cadherins are inextricably linked with catenins and form cadherin-catenin complexes which are critically important for cell-to-cell adhesion. However, in the course of carcinogenesis, cadherins are often inactivated or functionally blocked, which enables the development and progression of the tumor (14, 15). Cadherins have been evaluated in various human malignancies, e.g. pancreatic carcinoma, melanoma, hepatocellular carcinoma, glioblastoma, breast tumor and gastric cancer (14, 16). In numerous studies, E-cadherin has been described as a tumor suppressor (17, 18). However, according to more recent studies E-cadherin, particularly in late-stage tumors, can also promote cell migration and invasion, and even cancer progression (19, 20).

The action of catenin is not limited to the E-cadherin-dependent regulation of intercellular adhesion, it is also an important mediator of the Wnt signaling pathway. An increased level of β-catenin expression and its localization in the cell nucleus always induces carcinogenic features and promotes the proliferation and survival of cancer cells (21, 22). Saito et al. showed weak expression of E-cadherin only in 3 out of 16 seminomas in contrast to β-catenin, which was expressed in most cases. Similar results are presented by Guerra et al. (23, 24).

Galectin-3 belongs to the galectin family and has many important biological functions, such as: apoptosis, cell growth, pre-mRNA assembly, angiogenesis, differentiation and transformation. This lectin is mainly found in the cytoplasm, but can also be found in the nucleus, and is also secreted into body fluids such as serum and urine (25). Elevated serum galectin-3 levels have been reported in patients with colorectal and bladder cancer (26, 27). In patients with prostate cancer, serum galectin-3 levels have been shown to positively correlate with prostate-specific antigen, especially in the early clinical stage (28). Studies have shown that galectin-3 can be a diagnostic or prognostic biomarker of many diseases, including cancer. Research by Kayser et al. and Devouassoux-Shisheboran et al. showed that galectin-3 could potentially become a predictor of tumor aggressiveness and survival of patients with testicular cancer (29, 30).

The incidence of seminoma increases from year to year. New factors associated with cancer progression are still being investigated, and the results obtained are inconclusive. E-cadherin, B-catenin and galectin-3 may prove to be important biomarkers that have not yet been jointly evaluated in this type of cancer. The aim of the study is the immunohistochemical identification and evaluation of the expression of E-cadherin, B-catenin and galectin-3 in human testicular seminoma.

## 2 Materials and methods

### 2.1 Sample collection

The research was carried out on archival postoperative material collected from thirty patients of the Department of Urology of Medical University of Bialystok, operated on for testicular cancer in the years 2014-2022. The study protocol was approved by the Bioethics Committee of the Medical University of Bialystok (R-I-002/282/2019) and prior written informed consent was obtained from each subject.

The research material consisted of fragments of seminomas obtained during radical orchidectomy. The comparative material consisted of fragments of the surrounding unchanged tissue of the testis. All seminoma lesions were at the same pT1 stage were confined to the testis and epididymis without vascular/lymphatic invasion. Following surgery samples were immediately fixed in Bouin’s solution and routinely embedded in paraffin or placed in RNAlater solution (AM7024 Thermo Fischer) and stored in -80°C.

The paraffin blocks were cut into 4 µm section. Histological examinations were performed on haematoxylin and eosin-stained tissue sections. Immunohistochemical reactions were performed to detect β-catenin, E-cadherin and Gal-3. The material stored in the RNAlater solution was subjected to real-time PCR to evaluate the expression of the genes encoding β-catenin, E-cadherin and Gal-3. The slides were evaluated using an Olympus BX43 optical microscope with a built-in digital camera and connected to a computer. Color microscopic images of β-catenin, E-cadherin and Gal-3 at 200x magnification (at least 5 fields from each slide of a given patient) were archived and saved in jpg format on a computer hard drive.

### 2.2 Immunohistochemistry

Immunostaining was made by the following protocol (detailed described in Kasacka et al. (2018) (31): paraffin-embedded sections were deparaffined and hydrated in pure alcohols. The sections of testicular tissue were subjected to pretreatment in a pressure chamber and heated using Target Retrieval Solution Citrate pH=6.0 (Agilent Technologies, Inc. Santa Clara, CA, USA). After cooling down to room temperature, the sections were incubated with Dako REAL Peroxidase-Blocking Solution (Agilent Technologies, Inc. Santa Clara, CA, USA). The sections with the primary antibodies: β-catenin (1:2000; ab32572, Abcam, Cambridge, UK), E-cadherin (1:500; ab76055, Abcam, Cambridge, (UK) and galectin-3 (1:1000; MA1-940, Invitrogen) were incubated 24 hours at +4°C in a humidified chamber. Procedure was followed by incubation with secondary antibody (*REAL™ EnVision™ Detection System, Peroxidase/DAB, Rabbit/Mouse detection kit* (K5007; Agilent Technologies Denmark Ap/S, Produktionsvej 42, 2600 Glostrup, Denmark). The bound antibodies were visualized by incubation with DAB Flex chromogen. Finally the testicular tissue sections were counterstained in hematoxylin QS (H-3404 Vector Laboratories, Burlingame, CA, USA) and observed under a light microscope. Sections with seminoma tissues were dehydrated and the specificity of the antibodies was confirmed using a negative control, where the antibodies were replaced by Antibody Diluent (S3022; Agilent Technologies Denmark Ap/S, Produktionsvej 42, 2600 Glostrup, Denmark). The negative control showed no apparent immunoreactivity in the seminiferous tubules.

### 2.3 Real-time PCR

Seminoma and normal tissue samples taken from the material after operation were placed in an RNA-later solution. Total RNA was isolated using NucleoSpin^®^ RNA Isolation Kit (Machery-Nagel). Quantification and quality control of total RNA was determined using a spectrophotometer - NanoDrop 2000 (ThermoScientific). An aliquot of 1 µg of total RNA was reverse transcribed into cDNA using iScript™ Advanced cDNA Synthesis Kit for RT-qPCR (BIO-RAD). Synthesis of cDNA was performed in a final volume of 20 μl using an Thermal Cycler (Model SureCycler 8800, Aligent Technologies). For reverse transcription, the mixtures were incubated at 46°C for 20 min, then heated to 95°C for 1 min and finally cooled quickly at 4°C. Quantitative real-time PCR reactions were performed using Stratagene Mx3005P (Agilent Technologies) with the SsoAdvanced™ Universal SYBER^®^ Green Supermix (BIO-RAD). Specific primers for E-cadherin, β-catenin and GAPDH (GAPDH) were designed by BIO-RAD Company. The housekeeping gene GAPDH (GAPDH) was used as a reference gene for quantification. To determine the amounts of levels of test genes expression, standard curves were constructed for each gene separately with serially diluted PCR products. PCR products were obtained by cDNA amplification using specific primers as follows: E-cadherin (qHsaCEP0049339, BIO-RAD), β-catenin (qHsaCID0010363, BIO-RAD), and GAPDH (qHsaCED0038674, BIO-RAD). QRT-PCR was carried out in a doublet in a final volume of 20 μl under the following conditions: 2 min polymerase activation at 95°C, 5 s denaturation at 95°C, 30 s annealing at 60°C for 35 cycles. PCR reactions were checked, including no-RT-controls, omitting of templates, and melting curve to ensure only one product was amplified. The relative quantification of gene expression was determined by comparing Ct values using the ΔΔCt method. All results were normalized to GAPDH.

### 2.4 Statistical analysis

All data were analyzed for statistical significance using the Statistica version 12.0 computer software package+e. The mean values were computed automatically; significant differences were determined by one-way ANOVA test; p < 0.05 was considered significant.

## 3 Results

In the present study, sections from thirty seminomas and normal tissues testis were examined. A total of 30 samples were considered for the study. The mean age of the patients at the time of operation was 37 years, with a range 23-63 years. A positive immunohistochemical reaction in the form of a brown stain indicated the presence of the tested antigen.

### 3.1 Immunohistochemical evaluation

**Figure 1:**
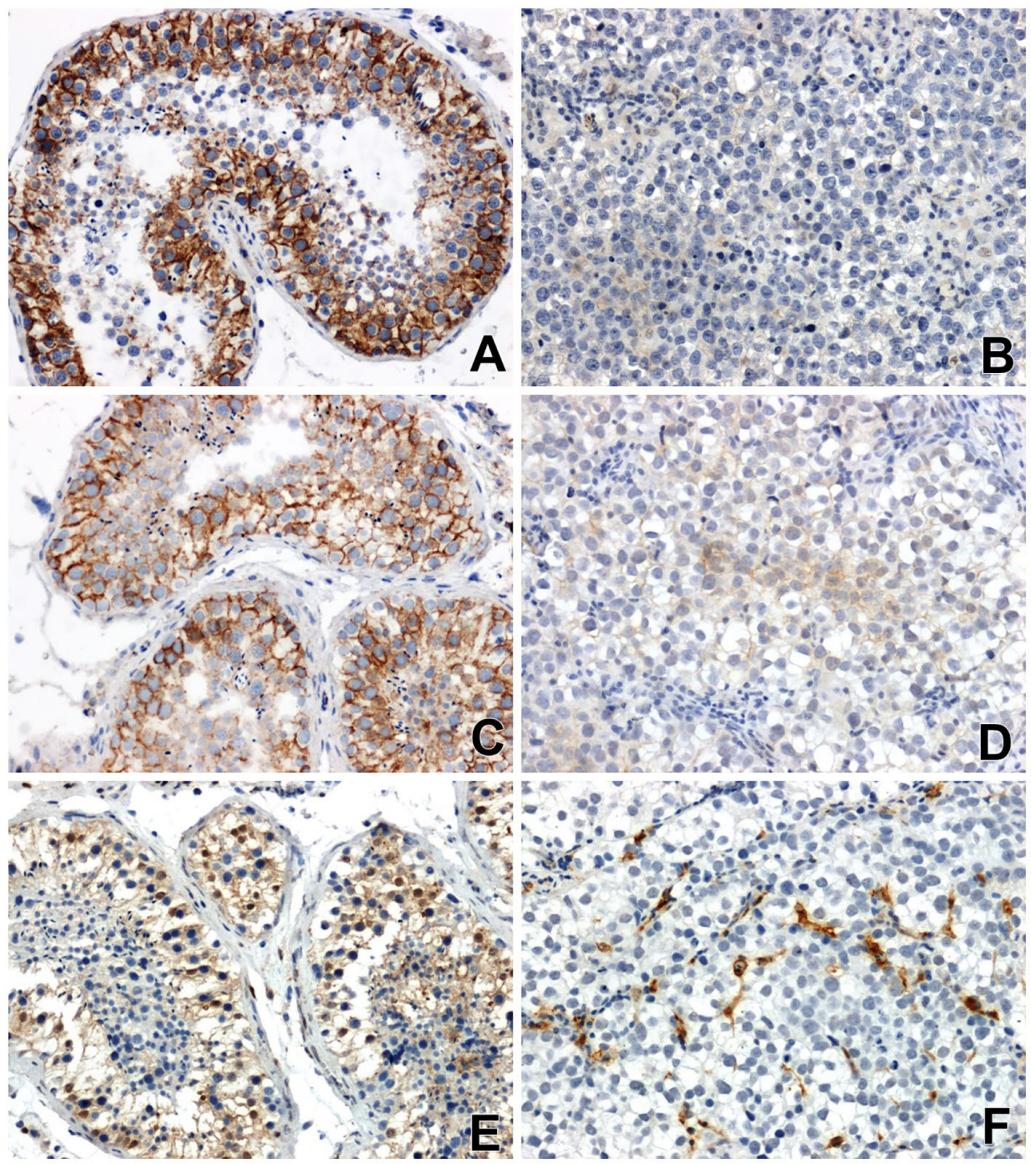
Immunoreactivity and localization in testicular tissues: of β-catenin **(A)** in normal non-neoplastic tissue, **(B)** in seminoma (very weak or negative reaction), of E-cadherin **(C)** in normal tissue, **(D)** in seminoma (a low specific signal) and Gal-3 **(E)** in normal tissue, **(F)** in seminoma, x200.

Strong β-catenin immunoreactivity was found in non-cancerous testicular tissue, mainly in the contact areas between epithelial cells of the convoluted tubules of the testis (**Figure 1A**).

The immunoreactivity of β-catenin in all seminomas was very low or negative. Nuclear localization of β-catenin or low-grade perinuclear expression was observed only in single tumor cells (**Figure 1B**).

Immunohistochemical analysis showed a clear immunoexpression of E-cadherin in non-neoplastic tissues of the testis. A positive result of the reaction, in the form of a colour reaction, was observed mainly in the membrane localization of the epithelial cells of the seminiferous tubules (**Figure 1C**). Meanwhile, E-cadherin immunoreactivity in seminomas was significantly attenuated and restricted to certain cell groups in membrane and cytoplasmic localization (**Figure 1D**).

Reaction with anti-antibody Gal-3 showed clearly positive immunoreactivity in the cytoplasm of the cells of the seminiferous tubules, as well as in the nuclei of some cells in the testis without signs of neoplastic transformation (**Figure 1E**). In seminomas, a clear positive reaction was observed in endothelial cells and single stromal cells (**Figure 1F**).

### 3.2 Real-time PCR

**Figure 2:**
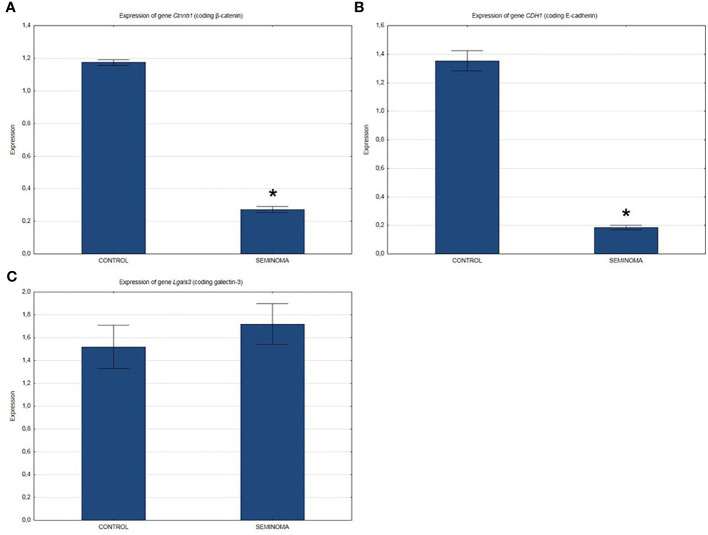
Expression of genes in normal and seminoma tissues encoding: **(A)** β-catenin, **(B)** E-cadherin, and **(C)** galectin-3. *p < 0.05 seminoma vs. control.

QRT-PCR analysis showed a significant decrease in the expression of β-catenin and E-cadherin genes (**Figures 2A, B**) and a slight increase in the expression of the Lgals3 gene encoding galectin-3 (**Figure 2C**) in the seminomas compared to healthy tissue.

## 4 Discussion

According to the latest epidemiological data, testicular cancer accounts for 1.6% of all cancers in men and the number of diagnosed cases is increasing every year. The dominant type of cancer is germ cell carcinomas, including seminomas and non-seminomas.

The exact etiology of seminoma is unknown. The last hypothesis is that endocrine-disrupting environmental factors contribute to the abnormal development of gonocytes. Expanding knowledge on the mechanisms of testicular cancer formation is necessary to continuously improve the effectiveness of prevention and treatment of this malignant tumor. Considering the important role of β-catenin, E-cadherin and galectin-3 in the process of carcinogenesis, it seems reasonable to conduct a study aimed at evaluating and comparing gene expression and activity of the above proteins in testicular seminoma compared to healthy tissue. Immunohistochemical tests and real-time PCR were used as research methods (32).

Searching the literature, we were able to find only individual articles on similar topics. Moreover, data describing changes in the biology of seminomas compared to normal testicular tissue are also limited (21, 24, 29, 30, 33–35).

In our study, we showed significant attenuation of immunoreactivity and expression of β-catenin and E-cadherin genes in seminoma compared to strong expression in control tissue. The study of gal-3 showed a change the localization of this protein. In seminomas, quite strong immunoreactivity was demonstrated in endothelial cells and connective tissue stroma, while in normal tissue, the presence of galactin-3 was observed in the cytoplasm of the epithelium of the seminiferous tubules and the intensity of the reaction was moderate.

In the presented study, 30 patients with organ-limited diagnosis of testicular seminoma, without features of distant metastases, were evaluated. The expression of genes encoding β-catenin and E-cadherin was significantly reduced, while in the case of the gene encoding galectin-3, a slight increase in expression was found in comparison to healthy tissue.

Changes in β-catenin immunoreactivity in seminomas compared to control tissue have been demonstrated in several previous studies. An immunohistochemical study by Guerra et al. showed weak expression of β-catenin in investigated seminomas. Low expression of E-cadherin and no activity of beta-catenin in cell nuclei were demonstrated (24). Chovanec et al. evaluated beta-catenin alone in testicular cancer. They showed a marked decrease in its expression in seminomas compared to other types of testicular tumors. They also proved that the intensity of beta-catenin expression may positively correlate with poor prognostic factors (33). Lobo et al. analyzed potential biomarkers for an increased risk of disease recurrence in the first stage. The results obtained by these researchers indicate that patients with elevated beta-catenin expression have a higher risk of recurrence of the disease at this stage (34).

There are few reports relating to E-cadherin in testicular seminoma. Saito et al. evaluated e-cadherin expression in 16 patients diagnosed with seminoma. E-cadherin was detected in only three patients (21). Honecker and his research team also evaluated E-cadherin in testicular cancer. It should be noted that E-cadherin expression was significantly lower in seminomas compared to non-seminomas (36).

There are few reports in the available literature on the importance and function of galectins in testicular seminoma. Kayser et al. analyzed lectins as a prognostic factor in testicular seminoma with lung metastases. Compared to the primary tumor, none of the analyzed features, including galectin-3, was stable and statistically significant in the metastatic lesion. On the basis of the results obtained by the authors of determined galectins, no clear conclusions can be drown as to their significance in the prognosis of testicular seminoma (29). Devouassoux-Shisheboran et al. using immunohistochemistry and RT-PCR, analyzed galectin-3 expression in testicular cancer. Galectin-3 expression, however, was demonstrated in seminomas, with no apparent significant difference compared to control tissue (30). In the available literature, we have not found a study evaluating all three factors related to cell adhesion in seminoma at the same time. It can therefore be assumed that these are the first studies of this type.

The functions of β-catenin as a co-regulator of the transcription process and a protein necessary for intracellular adhesion are well known. Under the control of activation by Wnt, β-catenin accumulates in the cytoplasm and then translocated to the nucleus, where it promotes the transcription of genes encoding mostly oncoproteins. Beta-catenin translocated to the nucleus binds to lymphoid stimulating factor (LEF) and T cell factor (LEF-1/TCF), to form a powerful transcription factor of genes such as c-Myc, c-Jun, CCND1, metalloproteases, cyclin D1, vimentin, etc., all related to cell proliferation, invasion and EMT (epithelial-mesenchymal transition) (Liu et al, 2022). In our study, we demonstrated very weak expression of β-catenin in the nuclei of only single seminoma cells. These results are consistent with several previously cited observations of β-catenin staining in testicular cancer (33, 34, 37).

The important milestone in the pathogenesis of GCNIS (Germ cell neoplasia in situ) is the failure of germ cell differentiation/maturation due to lack of adequate signals from the somatic niche during early fetal development, which should normally decrease the expression of pluripotency factors (38). The atypical gonocyte retains the expression of embryonic markers such as OCT3/4 and AP-2γ, which leads to the dissemination of seminomas. Neoplastic gonocytes advance as the tubular walls lose elasticity, filling and widening the tubules (intratubular neoplasia) until the disappearance of the barrier produces leakage and dispersion of neoplastic cells (solid seminoma). This mechanism is independent of the Wnt/Beta catenin pathway (38).

E-cadherin, due to its important role in maintaining balance in the process of cell adhesion, cell differentiation and growth, is a very important factor in the context of the process of carcinogenesis. Similar to β-catenin, E-cadherin expression follows the same pattern: high in control tissue, low in tumor tissue. Based on the β-catenin/E-cadherin relationship described above, this result fully confirms the relationship between the two proteins and proves that both β-catenin and E-cadherin in seminoma are likely to play an important role in the development of this tumor. A reduced level of elements of the E-cadherin-beta-catenin complex impairs cell differentiation, promotes the development of local advancement and distant metastasis. The obtained results are confirmed in the works of other authors mentioned above (35, 39).

Galactin-3 participates in many processes important from the oncological point of view, such as: cell growth, apoptosis, differentiation and angiogenesis. Galectin-3 in cancer patients induces secretion of IL-6, G-CSF, sICAM-1 and GM-CSF from blood vascular endothelial cells *in vitro* and in mice. These cytokines interact in an autocrine/paracrine manner with the vascular endothelium to increase the expressions of the endothelial cell surface adhesion molecules E-selectin, ICAM-1 and VCAM-1, resulting in increased endothelial adhesion of cancer cells and increased migration of endothelial cells, with formation of tubules and branch points (angiogenesis) (40). Our results demonstrated galectin-3 expression in both control tissue and seminoma. The difference consisted in a different location, in the healthy tissue it was the cytoplasm of the epithelial cells of the seminiferous tubules and nuclei of some single cells, in the cancerous tissue mainly the endothelium. Due to the small amount of scientific reports on galectin-3 in seminoma, it is not easy for us to refer and draw clear conclusions. Nevertheless, we consider the localization of galectin-3 in the endothelium in the seminoma as an obvious manifestation of the participation of the studied protein in the angiogenesis process in carcinogenesis.

In summary, our study showed significant differences in the immunoreactivity and expression of the assessed factors: β-catenin, E-cadherin and galectin-3 in testicular seminoma compared to control material. While the results concerning β-catenin and E-cadherin were not surprising, the immunoreactivity of galectin-3 and its localization in the seminoma seem to be the most interesting finding in our study. The results obtained in the study indicate a fairly significant role of the above-mentioned proteins in the process of carcinogenesis in testicular seminoma. They may indicate further aspects of the pathomechanism of seminomas formation and at the same time lead to a better understanding of the biology of these tumors. With the intensification of further research on seminomas, they could be a target of therapy. We hope that the information obtained will help in the future in determining new markers of this disease. This would allow earlier detection of the disease, and thus the implementation of appropriate treatment.

## Data availability statement

The original contributions presented in the study are included in the article/supplementary materials, further inquiries can be directed to the corresponding author.

## Ethics statement

The studies involving humans were approved by bioethics committee at the Medical University in Białystok. The studies were conducted in accordance with the local legislation and institutional requirements. The participants provided their written informed consent to participate in this study. Written informed consent was obtained from the individual(s) for the publication of any potentially identifiable images or data included in this article.

## Author contributions

GM: Conceptualization, Data curation, Investigation, Methodology, Writing – original draft. ND: Conceptualization, Data curation, Methodology, Writing – review & editing. IK: Conceptualization, Data curation, Formal Analysis, Methodology, Supervision, Writing – review & editing.
